# Pros and Cons of Ion-Torrent Next Generation Sequencing versus Terminal Restriction Fragment Length Polymorphism T-RFLP for Studying the Rumen Bacterial Community

**DOI:** 10.1371/journal.pone.0101435

**Published:** 2014-07-22

**Authors:** Gabriel de la Fuente, Alejandro Belanche, Susan E. Girwood, Eric Pinloche, Toby Wilkinson, C. Jamie Newbold

**Affiliations:** Institute of Biological Environmental and Rural Sciences, Aberystwyth University, Aberystwyth, Ceredigion, United Kingdom; Graz University of Technology (TU Graz), Austria

## Abstract

The development of next generation sequencing has challenged the use of other molecular fingerprinting methods used to study microbial diversity. We analysed the bacterial diversity in the rumen of defaunated sheep following the introduction of different protozoal populations, using both next generation sequencing (NGS: Ion Torrent PGM) and terminal restriction fragment length polymorphism (T-RFLP). Although absolute number differed, there was a high correlation between NGS and T-RFLP in terms of richness and diversity with R values of 0.836 and 0.781 for richness and Shannon-Wiener index, respectively. Dendrograms for both datasets were also highly correlated (Mantel test = 0.742). Eighteen OTUs and ten genera were significantly impacted by the addition of rumen protozoa, with an increase in the relative abundance of *Prevotella*, *Bacteroides* and *Ruminobacter*, related to an increase in free ammonia levels in the rumen. Our findings suggest that classic fingerprinting methods are still valuable tools to study microbial diversity and structure in complex environments but that NGS techniques now provide cost effect alternatives that provide a far greater level of information on the individual members of the microbial population.

## Introduction

Microbial populations that inhabit gut environments play an essential role in the wellbeing of the host by utilizing nutrients that otherwise are not digestible by the host, creating an environment that is not conducive for pathogen survival and stimulating the immune system [1]. To understand form and function of complex ecosystems identifying primary drivers of microbial diversity and community structure is essential [2]. The evolution of the study of rumen microbial diversity is similar to that of other microbial ecosystems, moving from culture-based and microscopic observations to the use of culture-independent, molecular techniques. The small subunit ribosomal RNA gene (S rRNA) is the most common target for characterising bacterial diversity in such environments [3]. The use of fingerprinting techniques can provide useful information on the structure of the rumen microbiome. The more commonly used techniques in the study of the rumen microbial ecosystem are single-strand conformation polymorphism [4], denaturing gradient gel electrophoresis [5], restriction fragment length polymorphism (RFLP) and its variant terminal-RFLP [6]. These methods have used non-targeted approaches to identify differences and similarities in microbial communities in response to differences in host species, diets and feed efficiency, [7], [8] but they do not provide direct sequence information. In spite of this shortcoming, fingerprinting techniques continue to be used as they provide a quick snapshot of the microbiota. They can show that there are differences between various treatments, but they cannot be used to identify key species in a system biology approach [9].

The development of next generation sequencing (NGS) technologies has supported a rapid growth of applications [10] including developments in the screening of complex microbial communities [11], [12]. In particular, the characterization of bacterial 16S rRNA gene pools through massively parallel amplicon sequencing is becoming a method of choice which can replace previously used clone library sequencing techniques [13] and potentially even fingerprinting techniques, such as T-RFLP. The increasing numbers, quality and length of reads per run, together with the possibility of “barcode-tagging” amplicons with sample-specific adaptors to allow samples to be multiplexed [14], provides the opportunity to screen multiple samples at high sequencing depth. However, the general reproducibility and robustness of NGS, its potential to adequately recover relative template abundances, and its comparability to other screening techniques like rRNA gene fingerprinting are still a matter of debate [10]. The respective literature is continuing to grow and provides both supportive [15] and less supportive arguments [16] regarding the use of NGS in microbial ecology studies. In addition to the generally accepted need for quality filtering to avoid overestimation of diversity [16], issues with the technical reproducibility and semi-quantitative potential of pyrotag sequencing have also been raised [17]. Fingerprinting techniques might thus still represent an acceptable and low-cost way to study microbial diversity in complex ecosystems. In this study we evaluated the use of the Ion Torrent PGM NGS as an alternative to fingerprinting techniques to study microbial diversity in gut environments by sequencing the V3 region of the 16S rRNA gene and comparing the results with those obtained using a terminal-RFLP fingerprinting study of the same gene (16S rRNA). To be able to establish a reliable comparison we used DNA from 8 animals during three different states of rumen protozoal colonization. Rumen protozoa are not essential to rumen function but can significantly affect ruminal fermentation and their host's nutrition [18], so the progressive colonization of rumen protozoa served as a powerful model to evaluate changes in the rumen microbiome of sheep.

## Methods

### Experimental design description, colonization procedure and collection of rumen samples

All animal procedures were carried our according to the Animals (Scientific Procedures) Act 1986 (PLL 40/316; PIL 40/9798) in accordance with the guidelines of the European Directive 2010/63/EU and after approval by the Aberystwyth University's Internal Ethical Review Panel. Eight mature protozoa-free Texel-crossbreed sheep isolated from faunated sheep at birth [19] and approximately 4 years old at start of experiment were used in an experiment with three consecutive periods, with a 3-month adaptation phase between each period. For the first period (P1) animals remained fauna-free; for the second period (P2) they were inoculated with a mixed holotrich population (*Isotricha intestinalis*, *Isotricha prostoma* and *Dasytricha ruminantium* species), obtained from cryopreserved samples from monofaunated sheep. Holotrich protozoa were inoculated by oral administration of 50 mL of holotrich protozoa mix diluted in Coleman's Simplex type Solution [20]. For the third period (P3) animals were inoculated with rumen fluid obtained from control animals (with a natural protozoal population consisting of the subfamilies Entodiniinae (87%) and Diplodiniinae (2.5%), *Epidinium sp.*(7%), *Isotricha sp.*(0.5%) and *Dasytricha sp.*(2%)). During the last month of each period sheep were kept in individual pens with free access to fresh water and mineral blocks and fed an experimental diet composed of 67% ryegrass hay and 33% ground barley to meet 1.5 times maintenance requirements [21]. Diet was distributed in two equal meals per day (0900 and 1900h). At the end of each period rumen fluid (about 350 mL per animal) was obtained by oesophageal tubing before the morning feeding. Then rumen fluid was filtrated trough 250 µm^2^ pore size nylon mesh and pH recorded.

### Biochemical analyse and protozoal counts

Rumen fluid samples were added to 20% orthophosphoric acid (containing 20 mM 2-ethyl butyric acid as an internal standard, 1 mL acid/4 mL of rumen fluid) to deproteinise the samples. SCFA analysis (acetate (C2), propionate (C3) and N-butyrate (C4) acids) was conducted after sedimenting for 24 h before being filtered through a Cronus 0.45 µm^2^ 25 mm Nylon Syringe Filter with prefilter into a glass vial and capped. SCFAs were determined by gas liquid chromatography using 2-ethyl butyric acid as the internal standard as described by [22]. Ammonia (NH3) concentration was determined using the phenol method of Whitehead [23].

Total protozoal counts were quantified by optical microscope using the procedure described by Dehority [24] and adapted by de la Fuente et al. [25]. Concentrations of *Isotricha sp.* and *Dasytricha sp.* were calculated as representative of holotrich protozoa, and of subfamily Entodiniinae, subfamily Diplodiniinae and *Epidinium sp.*, as representative of entodiniomorphid protozoa.

### Molecular analyses

#### DNA extraction

Rumen fluid samples were stored on ice until frozen at −80°C prior to freeze drying. Before extraction of nucleic acids, freeze-dried samples were disrupted by bead beating. Freeze-dried samples (100 mg) were added to a 2-mL screw top tube with one autoclaved glass bead added (4 mm, undrilled, G/0300/53, Fisher Scientific, UK). Samples were bead-beaten for 90 s at 5000 rpm in a Mini-Beadbeater (Biospec products Inc., Bartlesville, OK). DNA was then extracted using QIAGEN QIAamp DNA stool mini kits (Qiagen Ltd., UK) as previously described [26].

#### Fingerprinting analysis (T-RFLP)

PCR was performed using a 16S rRNA bacterial-specific primer pair, cyanine-labelled 27F (5′-AGA GTT TGA TCC TGG CTG AG-3′) and unlabelled 1389R (5′-AGG GGG GGT GTG TAG AAG-3′) [27] following Skøivanová et al. [26]. A 25-µl reaction was prepared containing 1.25 U GoTaq DNA polymerase (Promega UK Ltd., Southampton, UK), 1× Promega reaction buffer, 1.75 mM MgCl_2_, 0.2 mM of each dNTP with each primer used at 0.5 µM. Resultant amplicons were analysed on a 1% (w/v) TAE agarose gel to assess the quality of amplification.

DNA concentration of each amplified and purified sample was determined by spectrophotometry (Nanodrop ND-1000 spectrophotometer) to enable a standardised quantity of 50 ng DNA for digestion with restriction enzymes. Digestion of samples was carried out using the restriction enzymes, HhaI, HaeIII, RsaI and MspI (New England Biolabs UK Ltd.) following the manufacturers recommendations with the exception of HhaI where the recommended addition of bovine serum albumin was omitted.

Restriction digests (20 µL) were purified by ethanol precipitation in a thermowell 96-well PCR plate (Costar; Corning Inc., NY). DNA was precipitated with 120 µL of 95% ethanol at −80°C, 4 µL EDTA (100 mM), 4 µL sodium acetate (3M, pH 5.2) and 4 µL of glycogen (20 mg/ml) and 30 min centrifugation at 4°C at 3000 ***g***. DNA pellets were washed twice with 200 µL of 70% ethanol, air-dried at room temperature and re-suspended in 35 µL sample loading solution buffer including a 600 bp size standard (Beckman Coulter Inc., Fullerton). T-RFs were separated on a CEQ 8000 Genetic Analysis System (Beckman Coulter, High Wycombe, UK) using the Frag4 parameters (denaturation step at 90°C for 120 seconds; injection at 2 kV for 30 seconds; separation at 4.8 kV for 60 min with a capillary temperature of 50°C). The protocol and software used was as described by [26] using the Local Southern method to distinguish true peaks from background noise. In this instance the following criteria was applied prior to exporting data from the CEQ 8000 genetic analysis system: Slope threshold of 5 and relative peak height of 5% (where 5% of the second highest peak was used as the lower threshold for peak identification). These parameters allow detection and elimination of smaller, broader peaks that would have a less specific size and not be indicative of single true OTUs.

#### NGS analysis

Amplification of the V3 hyper variable region of 16S rRNA was carried out with primers 341F and 518R [28]. The forward primer (5′-CCTACGGGAGGCAGCAG-3′) carried the Ion Torrent Primer A-key adaptor sequence (5′-CCATCTCATCCCTGCGTGTCTCCGACTCAG-3′) and the reverse primer (5′-ATTACCGCGGCTGCTGG-3′) carried the Ion Torrent Primer P1-key adaptor sequence A (5′-CCTCTCTATGGGCAGTCGGTGAT-3′) followed by a 12 nucleotide sample specific barcode sequence (Table S1). For each sample replicate PCR was performed in duplicate; a 25-µl reaction was prepared containing 1.25 U FastStart High Fidelity Enzyme Blend, 10× FastStart High Fidelity Buffer with 18 mM MgCl_2_ (Roche Diagnostics Ltd., Burgess Hill, UK), 0.2 mM of each dNTP (Promega UK Ltd., Southampton, UK), 0.2 µM of each primer and 1 µl DNA template at 2.5–125 ng/µl. The conditions used were a hot start of 95°C for 10 min, 95°C for 2 min, followed by 22 cycles of 95°C for 30 s, 50°C for 30 s and 72°C for 30 s with a final extension at 72°C for 7 min. Reactions were amplified in a T100 thermal cycler (Bio-Rad, Hemel Hempstead, UK). Resultant amplicons were visualized on a 1% (w/v) TAE agarose gel to assess quality of amplification before pooling the duplicate reactions.

Pooled PCR reactions for all sample replicates were purified as per Roche technical bulletin 2011-007 (January 2012) ‘Short Fragment Removal Procedure for the Amplicon Library Preparation Procedure’ using Agencout AMpure XP beads (Beckman Coulter Inc., Fullerton, USA). DNA concentration of the purified PCR products was assessed using an Epoch Microplate Spectrophotometer with a Take3 Micro-Volume plate (BioTek UK, Potton, UK) to enable equi-molar pooling of samples with unique barcode sequences. Each library was further purified using the E-Gel System with E-Gel SizeSelect 2% Agarose gel (Life Technologies Ltd, Paisley, UK). Purified libraries were assessed for quality and quantified on an Agilent 2100 Bioanalyzer with a High Sensitivity DNA chip (Agilent Technologies UK Ltd, Stockport, UK). The sample libraries were subsequently sequenced using the Ion Torrent PGM sequencer following the Ion PGM Template OT2 200 Kit (Life Technologies Ltd, Paisley, UK).

The emulsion PCR was carried out using the Ion PGM Template OT2 200 Kit (Life Technologies) as described in the appropriate user Guide (Catalog number: 4480974, Revision 4.0) provided by the manufacturer. Sequencing of the amplicon libraries was carried out on the Ion Torrent Personal Genome Machine (PGM) system using the Ion PGM Sequencing 200 Kit v2 (all Life Technologies) following the corresponding protocol (Catalog number: 4482006, Revision 1.0). Raw sequence reads of all samples were deposited at the EBI Short Read Archive (SRA) from the European Nucleotide Archive (ENA) and can be accessed under the study accession number PRJEB5190.

Following sequencing, data were combined and sample identification numbers assigned to multiplexed reads using the MOTHUR software environment [29]. Data were denoised, low quality sequences, pyrosequencing errors and chimeras were removed, then sequences were clustered into OTU's at 97% identity using the CD-HIT-OTU pipeline (available from http://eeizhong-lab.ucsd.edu/cd-hit-otu, [30]). OTU's containing fewer than 10 reads were excluded due to the likelihood of them being a sequencing artifact. Samples were normalised by randomly resampling to the lowest number of sequences per sample (period/animal combination) using Daisychopper (www.genomics.ceh.ac.uk/GeneSwytch/). Taxonomic information on 16S rRNA transcripts was obtained by comparison against The Ribosomal Database Project- II (RDP) [31]. This method is widely used and provides rapid taxonomic classifications from domain to genus of both partial and full-length rRNA gene sequences. We considered only annotation with a bootstrap value over 0.7, stopping the assignation at the last well identified phylogenetic level and leaving successive levels as unclassified.

### Statistical analyses

Analysis of the TRFs was performed using Minkowski Metrics, Manhattan distances and unweighted pair group method with arithmetic mean (UPGMA). UPGMA was carried out using Neighbor within the Permanova+ package (version 1.0.2; primer-E, Ivybridge, UK). Permutational multivariate analysis of variance (Permanova) and canonical analysis of principal coordinates (CAP) [32] were also carried out using Permanova+. These analyses utilised Manhattan distances of fourth-root transforms of both T-RFLP and OTU abundance data. Permanova and CAP were performed using 9999 unrestricted permutations.

Data were prepared and tables and figures produced using Microsoft Excel and the ‘R’ software environment (version 2.15; http//www.r-project.org/). Richness and Shannon-Wiener diversity indices were calculated using normalised data as recommended to reduce over-inflation of true diversity in pyrosequencing data sets [33]. Species richness and Shannon-Wiener diversity were then analysed by one-way ANOVA using R. Spearman's product-moment correlations were performed between fermentation parameters (pH, NH_3_, C2, C3 and C4) and biodiversity indexes values from both datasets (NGS and T-RFLP). A Canonical Correspondence Analysis (CCA) was also performed, including the fermentation parameters as constraining variables in the model. CCA is known to be a useful tool to explain the structure of a multivariate data table by using environmental variables, assuming a unimodal distribution of “species” (OTUs or T-RFs) [34]. Thus, the ordination diagram represents not only a pattern of community distribution, but also the main features of the distribution of species along the environmental variables.

In an attempt to normalize the data, a square root transformation was used before analyzing the effect of the colonization of rumen protozoa on each individual OTU by ANOVA. To minimize the false discovery rate when pairwise comparison were made, P values were adjusted using the method of Benjamini and Hochberg [35] where significance was set at Q<0.1. Furthermore, only OTUs with an average abundance of 0.01% or higher were considered.

## Results

### Protozoal counts

Counts of total and groups of protozoa are shown in Table 1. No protozoa were found in any of the animals from P1. No significant differences were observed in the protozoa concentration between animals over the studied periods (P>0.05). Protozoal proportions in P2 consisted of an average of 23% and 77% *Isotricha sp.* and *Dasytricha sp.* respectively. Both *Isotricha spp* and *Dasytricha sp.* numbers decreased when a full protozoal fauna was established (Table 1, P<0.05). The protozoal population in P3 consisted of 89.3% subfamily Entodiniinae, 51% subfamily Diplodiniinae, 2.6% *Epidinium sp.*, 2.4% *Daystricha sp.* and 0.7% *Isotricha sp*.

**Table 1:** Protozoa concentration (log (numbers +1) per mL of rumen fluid) from sheep either protozoa-free (P1), faunated with holotrich protozoa (P2) or with a complete protozoal population (P3).

Protozoa	P1	P2	P3	SED	*P*
Isotricha sp	0^c^	4.02^a^	3.60^b^	0.120	<.001
Dasytricha sp	0^c^	4.58^a^	4.17^b^	0.086	<.001
Subf. Entodiniinae	0^b^	0^b^	5.80^a^	0.050	<.001
Subf. Diplodiniinae	0^b^	0^b^	4.55^a^	0.075	<.001
Epidinium sp.	0^b^	0^b^	4.12^a^	0.096	<.001
Total Protozoa	0^c^	4.69^b^	5.85^a^	0.061	<.001

Different superscript letters denote significant differences.

### Fermentation parameters

Changes in the fermentation pattern were observed in the three periods studied (Table 2). Concentration of ammonia increased when total protozoal fauna was present in the rumen of the sheep (P<0.001). A shift in the SCFA production was also observed, with an increase in the levels of acetic and butyric acids when rumen was colonized with protozoa (P2 and 3, P<0.01).

**Table 2:** Fermentation parameters of rumen samples from sheep either protozoa-free (P1), faunated with holotrich protozoa (P2) or with a complete protozoal population (P3).

	P1	P2	P3	SED	P
pH	6.94^a^	6.89^a^	6.73^b^	0.0496	0.002
NH3-N (mg/dL)	1.29^b^	1.39^b^	4.85^a^	0.583	<0.001
C2 (mM)	51.59^b^	60.16^a^	63.08^a^	2.36	<0.001
C3 (mM)	17.35	14.2	16.55	2.154	0.344
C4 (mM)	6.37^b^	9.6^a^	11.38^a^	1.159	0.002

Different superscript letters denote significant differences.

### T-RFLP dataset

For the fingerprinting analysis, 811 fragments from 4 different enzymes were obtained after filtering them together. The T-RFLP analysis included T-RFs in the range of 56 to 644 bp.

### NGS dataset

Three million, seven hundred and three thousand, seven hundred and forty eight sequences of average length 229 bp were obtained from the Ion Torrent PGM sequencing. Quality filtering resulted in 1,104,458 high quality sequences that were clustered into 864 unique OTUs with 18361 sequences per sample and period after normalisation. Rarefaction curves calculated from non-normalised data (Figure S1) showed that for each sample the corresponding curve did not plateau, indicating that complete sampling of these environments was not achieved. Good's coverage is an estimator of sampling completeness and calculates the probability that a randomly selected amplicon sequence from a sample has already been sequenced. At the 97% similarity level, all V3 samplings had more than 99.5% coverage, which means that over 200 (1/(1−0.995)) extra reads would be needed to detect a new phylotype.

### Diversity

The microbiome diversity within the eight animals between the three periods is shown in Table 3.

**Table 3:** Richness (Ri) and Shannon-Wiener (Sh) indexes of NGS (OTU matrix from Ion Torrent data) or T-RFLP (peaks in the amplicons of 16S rRNA gene digested using HhaI, HaeIII, MspI and RsaI.

	indexes	P1	P2	P3	SED	P
T-RFLP	Ri	172.2^b^	236.5^a^	166.5^b^	12.07	<0.001
	Sh	4.06^b^	4.68^a^	3.91^b^	0.121	<0.001
NGS	Ri	370^b^	514^a^	383^b^	39.8	0.005
	Sh	3.79^b^	4.59^a^	3.96^ab^	0.265	0.023

Genomic DNA was obtained from rumen samples from 8 animals (A1 to A8) either protozoa-free (P1), faunated with holotrich protozoa (P2) o with a complete protozoal population (P3). Different superscript letters denote significant differences.

**Figure 1:**
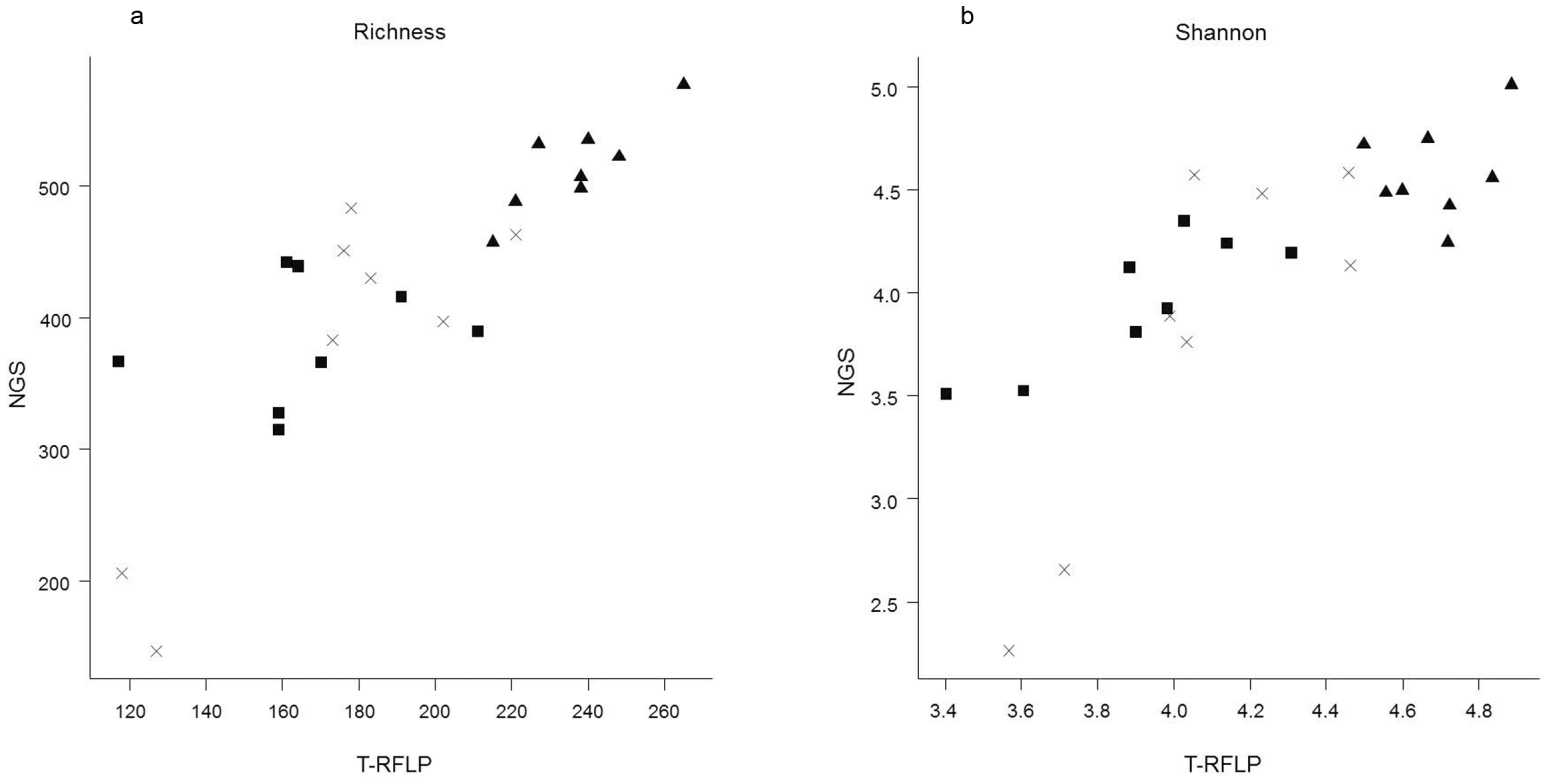
Correlation between T-RFLP and NGS. Correlation plot between NGS (OTU matrix from Ion Torrent data) and T-RFLP (peaks in the amplicons of 16S rRNA gene digested using HhaI, HaeIII, MspI and RsaI (dendrogram shows amalgamation of data from all four enzymes) on both Richness (Figure 2a) and Shannon-Wienner index (Figure 2b). Genomic DNA was obtained from rumen samples from 8 animals (A1 to A8) either protozoa-free (P1, crosses), faunated with holotrich protozoa (P2, black triangles) o with a complete protozoal population (P3, black squares).

**Figure 2:**
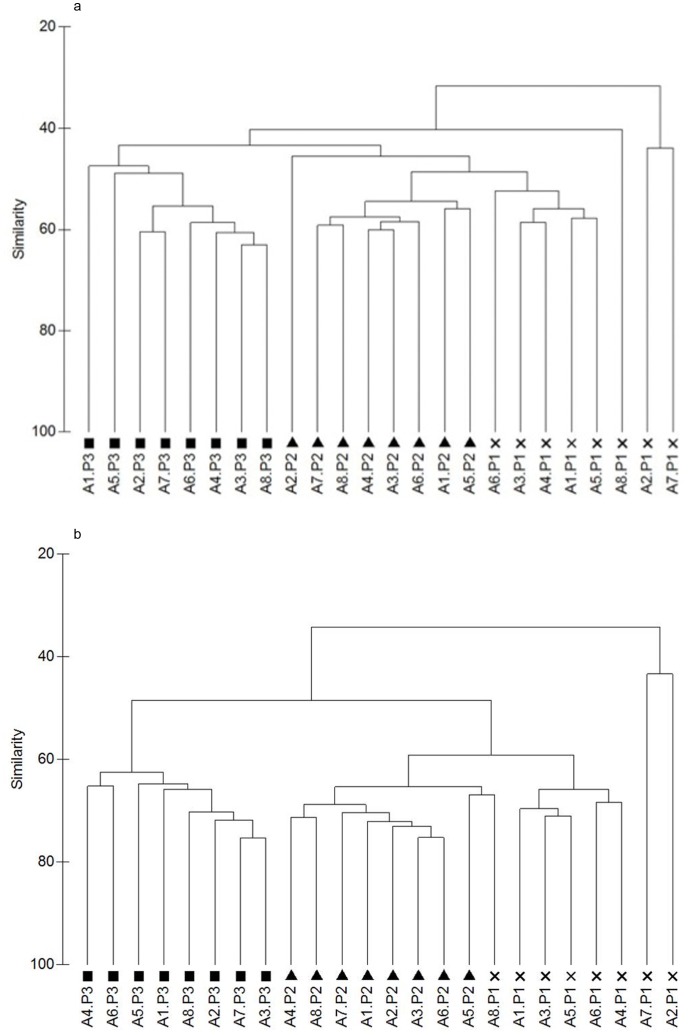
Dendrogram of the rumen bacterial population analysed by T-RFLP(a) or NGS(b). Dendrogram using the combination of Manhattan distances and UPGMA (unweighted pair group method with arithmetic mean) of T-RFLP (peaks in the amplicons of 16S rRNA gene digested using HhaI, HaeIII, MspI and RsaI (dendrogram shows amalgamation of data from all four enzymes) or NGS (OTU matrix from Ion Torrent data). Genomic DNA was obtained from rumen samples from 8 animals (A1 to A8) either protozoa-free (P1, crosses), faunated with holotrich protozoa (P2, black triangles) o with a complete protozoal population (P3, black squares).

**Figure 3:**
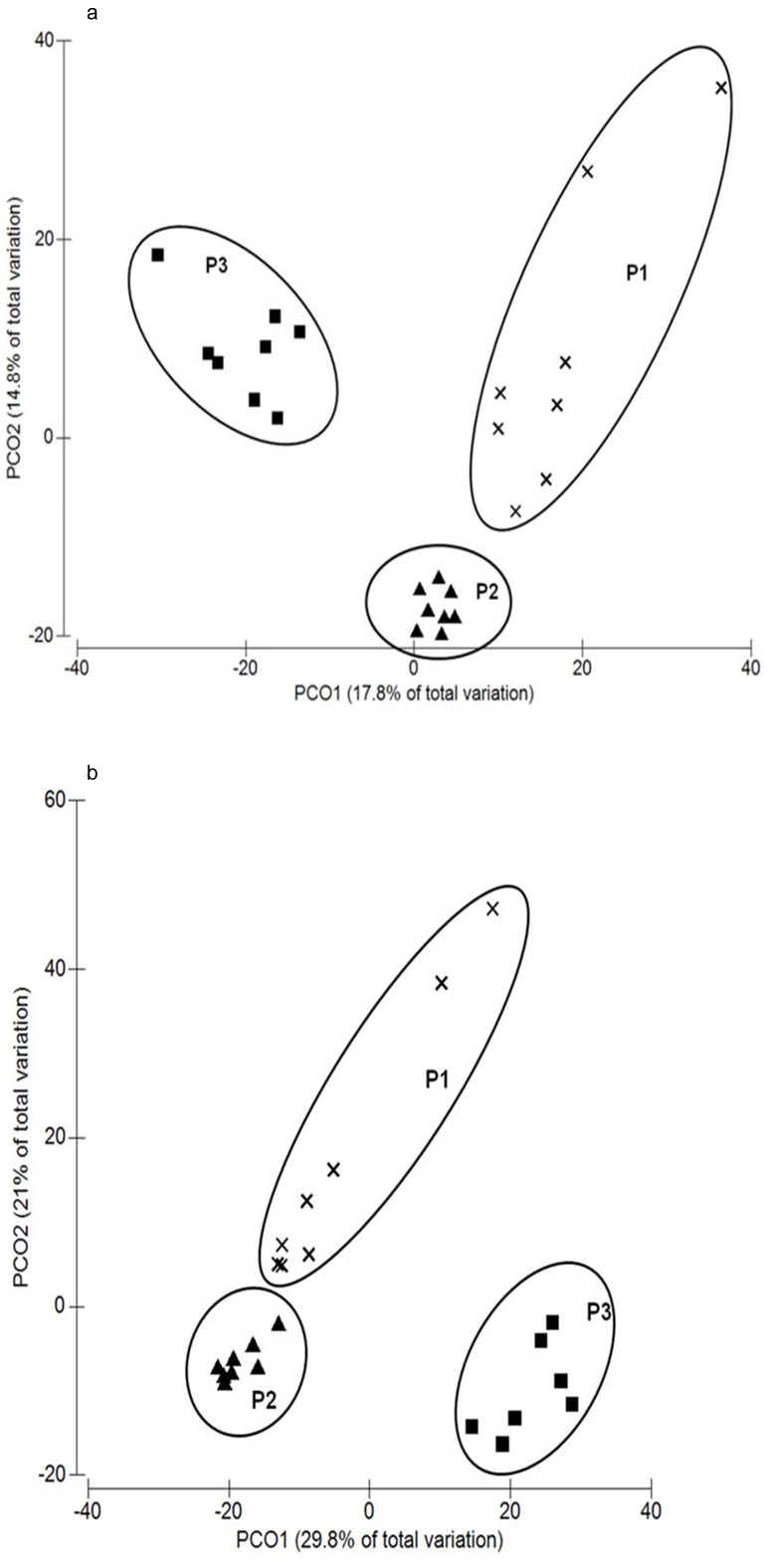
PCA plot of the rumen bacterial structure analysed by T-RFLP(a) or NGS(b). Principal Component Analysis of T-RFLP (Figure 4a, peaks in the amplicons of 16S rRNA gene digested using HhaI, HaeIII, MspI and RsaI (dendrogram shows amalgamation of data from all four enzymes) or NGS (Figure 4b, OTU matrix from Ion Torrent data). Genomic DNA was obtained from rumen samples from 8 animals either protozoa-free (P1, black crosses), faunated with holotrich protozoa (P2, black triangles) or with a complete protozoal population (P3, black squares).

Richness diversity index was lower in the results from T-RFLP than those obtained from NGS, although this didn't lead to large differences in the Shannon-Weiner index (P>0.05). Both richness abundance and Shannon-Wiener index from T-RFLP and NGS data were compared by correlation (Figures 1a and 1b). Correlation between both datasets showed an R value of 0.836 and 0.781 for richness abundance and Shannon-Wiener index, respectively. Dendrograms using the combination of Manhattan distances and UPGMA (unweighted pair group method with arithmetic mean) of NGS and T-RFLP datasets were also performed (Figures 2a and 2b). Mantel test was performed between both similarity matrices, resulting in a correlation of 0.742 (Monte-Carlo test, p = 0.001). When exploring the data by principle component analysis (Figures 3a and 3b), the same pattern was observed according to the period in both datasets (T-RFLP and NGS). Principal Components 1 and 2 together accounted for 32.6 and 50.8% of the variance within the data from T-RFLP and NGS, respectively.

### Correlation between Diversity and Fermentation parameters

Pearson's product-moment correlations were performed between fermentation parameters (pH, C2, C3, C4 and NH_3_) and Richness values from both datasets (NGS and T-RFLP, Table 4). Richness and Shannon index were negatively correlated with the presence of C3 in both datasets (P<0.05). None of the rest of the fermentation parameters showed significant correlation with the diversity indexes although correlation values showed little differences between both datasets (Table 4).

**Table 4:** Spearson's correlation (rho value) between Richness (Ri) and Shannon-Wiener (Sh) indexes of NGS (OTU matrix from Ion Torrent data) or T-RFLP (peaks in the amplicons of 16S rRNA gene digested using HhaI, HaeIII, MspI and RsaI) and fermentation parameters of rumen samples.

	Ri				Sh			
	NGS	P	T-RFLP	P	NGS	P	T-RFLP	P
pH	0.34	0.103	0.35	0.094	0.36	0.080	0.41	0.047
NH3	−0.24	0.249	−0.25	0.244	−0.22	0.308	−0.30	0.158
C2	0.18	0.405	0.08	0.722	0.12	0.567	−0.01	0.976
C3	−0.49	0.016	−0.48	0.017	−0.47	0.023	−0.50	0.014
C4	−0.26	0.227	−0.30	0.152	−0.30	0.148	−0.34	0.105

P values indicate statistical significance of the correlation.

**Figure 4:**
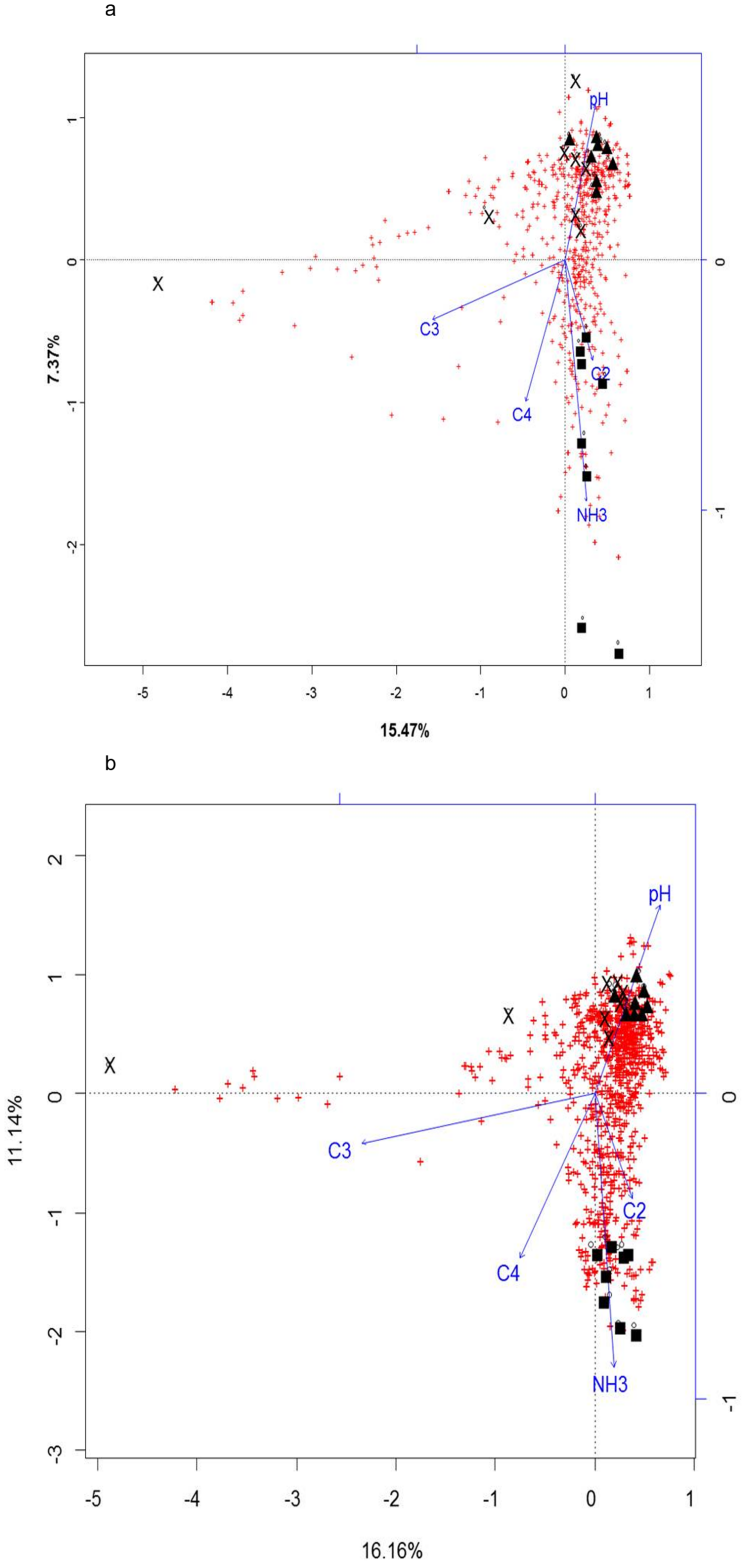
CCA plot of rumen bacterial communities analysed by T-RFLP(a) or NGS(b) and considering fermentation parameters. Canonical correspondence analysis of NGS (OTU matrix from Ion Torrent data) or T-RFLP (peaks in the amplicons of 16S rRNA gene digested using HhaI, HaeIII, MspI and RsaI. The formula used in the analysis was the following y (either NGS or T-RFLP data) = pH+Ammonia (NH3)+Acetate (C2)+Propionate (C3)+Butyrate (C4). Genomic DNA was obtained from rumen samples from 8 animals either protozoa-free (P1, black crosses), faunated with holotrich protozoa (P2, black triangles) o with a complete protozoal population (P3, black squares). Blue vectors indicate the effect of the constraining variables (pH, Ammonia (NH3), Acetate (C2), Propionate (C3) and Butyrate (C4).

Canonical Correspondence Analysis between TRF's or OTU's and fermentation products is shown in Figures 4a and 4b. A permutation test (199 permutations) was conducted and the model found to be highly significant (P = 0.005 in both dataset). The two main axes accounted together for 21.5 and 25% of total variation in T-RFLP and NGS datasets, respectively. The constraining variables explained 34.2% and 38.7% of the variability, respectively.

### Classification of NGS data

**Figure 5:**
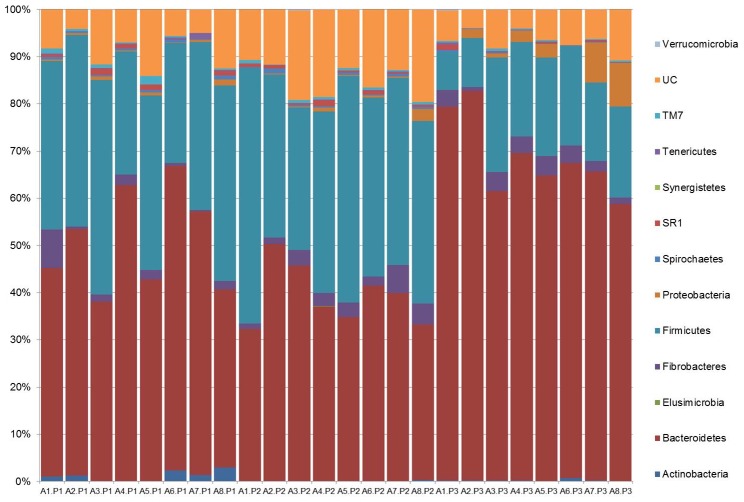
Phyla-level classification of OTUs from NGS at 97%. Phyla-level classification of OTUs from NGS at 97%. Genomic DNA was obtained from rumen samples from 8 animals (A1 to A8) either protozoa-free (P1), faunated with holotrich protozoa (P2) o with a complete protozoal population (P3).

Based on classification by RDPII, differences between the three periods were observed within the main phyla present (Figure 5, Table 5). In P1 and 3, Bacteroidetes was the most dominant phyla (49.7 and 68.5%, respectively), followed by Firmicutes (35.9 and 17.5%). In P2 Firmicutes was the most dominant phyla (40.2%), followed by Bacteriodetes (39.3%). When total protozoa were present, the proportion of Bacteroidetes increased significantly. (P<0.001, Table 5).

**Table 5:** Relative abundance (%) of the main phyla identified in rumen fluid from protozoa-free (P1), faunated with holotrich protozoa (P2) o with a complete protozoal population (P3) animals.

Phyla	P1	P2	P3	SED	*P*
Actinobacteria	1.15^a^	0.09^b^	0.24^b^	0.304	0.008
Bacteroidetes	49.7^b^	39.3^c^	68.5^a^	3.74	<.001
Elusimicrobia	0.00	0.01	0.00	0.006	0.283
Fibrobacteres	2.14	3	2.92	0.995	0.644
Firmicutes	35.9^a^	40.2^a^	17.5^b^	3.8	<.001
Proteobacteria	0.51^b^	0.6^b^	3.23^a^	0.949	0.019
Spirochaetes	0.49^a^	0.44^a^	0.16^b^	0.109	0.02
SR1	0.62	0.64	0.28	0.216	0.208
Synergistetes	0.00	0.01	0.00	0.002	0.06
Tenericutes	0.30	0.06	0.12	0.109	0.095
TM7	0.61	0.51	0.26	0.157	0.106
Verrucomicrobia	0.00	0.04	0.04	0.033	0.451

Superscripts show significant differences between means (P<0.05).

### Significant OTUs and Genera analysis

Eighteen OTUs with an average abundance of 0.1% or higher presented a significant variation in their abundances across the three experimental periods (Table S2, Corrected p-value<0.1). Among them, only three were classified to genus level (all the three belonged to *Prevotella sp.*) and 10 out from 18 belonged to the order Bacteroidales. Three of them were considered unclassified up to phyla level.

**Figure 6:**
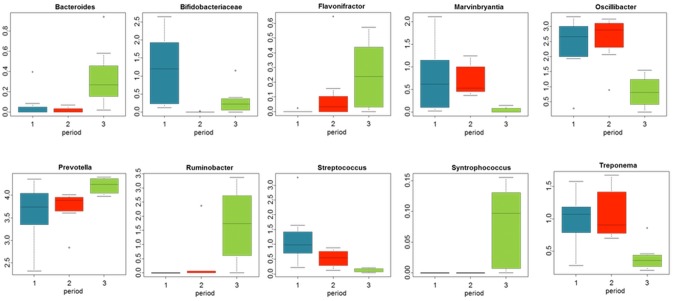
Bacterial genera significantly impacted by rumen protozoa. Boxplots of bacterial genera found to shift in their relative abundance (as percentage) when progressive colonization of rumen protozoa is applied. Samples come from rumen fluid from protozoa-free (P1), faunated with holotrich protozoa (P2) o with a complete protozoal population (P3) animals.

Ten out of forty two classified bacterial genera were impacted by colonization of the rumen by protozoa (Benjamini and Hochberg Q<0.1, Figure 6). Four of them (*Ruminobacter*, *Prevotella*, *Oscillibacter* and *Streptococcus*) were present at an average abundance higher than 0.5%.

## Discussion

High-throughput sequencing has allowed biologists to explore new ways to study sequence-based profiling and metagenomics in complex microbial communities, including those associated with human health and disease [36], [37]. With next generation sequencing platforms rapidly evolving, sequencing could be a regular reliable and price competitive alternative to classic fingerprinting methods. The only limitation of most NGS platforms is the short read lengths of approximately 250–400 bp that provide poor phylogenetic information as compared to full length 16S rRNA gene sequences (∼1500 bp). Although some platforms already include long lengths [38]. The cost of next generation sequencing has dropped dramatically over the last few years, and is becoming an affordable alternative to the more classical techniques to study microbial diversity [38], [39]. The use of barcodes to multiplex samples in NGS can reduce the cost per sample because most of the cost derived from NGS is associated to the price of the chip and the sequencing kits. In our own lab, the cost of processing 24 samples by Ion Torrent using one 316 chip was comparable at around £600, to characterisation by T-RFLP using four enzymes (circa £270). T-RFLP has been considered the default method to study microbial diversity in complex environments [40], [41], here we show NGS gives very similar and comparable information. However, NGS also allowed us to classify the bacterial populations that are affected by the presence of rumen protozoa in more depth than the data obtained with T-RFLP and target potential bacterial species responsible for metabolic shifts. Several web based tools such as phylogenetic assignment tool (PAT), TRUFFLER, APLAUS are available to determine microbial community composition by comparison with T-RFs predicted from an *in silico* analysis of rRNA database sequences [42], [43], [44] but the identification is still laborious and less accurate than the data obtained by NGS. The growing interest in NGS has attracted many experts from different disciplines and revolutionized the field of microbial ecology, promoting multiple research lines. This “revolution” has promoted the creation of numerous bioinformatic tools, that are available to process and analyse NGS data, as has been reviewed recently [39], [45]. Continuing improvements in data analysis algorithms applied to the NGS data has decreased the error rate of sequencing data bases that makes the technique more reliable than few years ago [46].

Comparison between both datasets showed a high correlation in the dendrograms (R = 0.742), richness abundance (R = 0.836) and Shannon-Wiener index (R = 0.781). PCA from both datasets (Figures 3a and 3b) showed a similar grouping effect by period that was highly significant (MonteCarlo test, P<0.001 in both datasets). Several diversity indices can be calculated to more objectively assess the effect of diet or location on the dominance among bacterial phylotypes [47]. The Shannon diversity index [48], which uses both the number and relative intensities of bands, has been calculated in several studies to test the effect of factors such as diet, sample processing methodology and defaunation on the rumen bacterial and archaeal community structure [49], [50], [51]. The Shannon diversity index reflects the diversity of abundant sequence types. In our study, Shannon indexes were similar between the two studied datasets (Table 3). In the first period a low value in the Shannon index was observed in the NGS dataset that could be matched to a low diversity in animals 2 and 7. This effect was also observed in the species richness in these individuals, and also in both dendrogram and PCA analysis (Figures 2a, 2b, 3a and 3b). In general, higher variability in the bacterial community was observed between individuals in P1 compared with P2 and P3 (standard deviation in Shannon index of NGS data of 0.88, 0.23 and 0.32 in P1, P2 and P3 respectively). These results are in accordance with the higher variability observed in fauna-free animals in the three most abundant phyla classified (Fibrobacteres, Firmicutes and Bacteroidetes) and suggest that the presence of ciliate protozoa in the rumen may have a stabilizing effect on the bacterial communities [52]. The data analysed in this study allowed us to investigate the transition of the rumen ecosystem when the progressive colonization of rumen protozoa was induced. This process is of great biological interest [53], because the presence of rumen protozoa has been associated directly or indirectly with metabolic processes, like the recycling of microbial N in the rumen, the production of methane, as well as changes in the short chain fatty acids profile produced by the rumen microbiome [54], [55], [56], [57].

Ten bacterial genera where significantly impacted by the presence of rumen protozoa (Figure 6). Among them, the increase of *Bacteroides*, *Prevotella* and *Ruminobacter* in the P3 indicates an increase on the proteolytic activity within the rumen when entodiniomorphid protozoa were present [58]. Holotrich protozoa contribute in a major way to the fermentation of soluble carbohydrates [59], but their role in proteolysis is more limited. In our study, presence of only holotrich protozoa did not alter the relative abundance of these N utilizing bacteria. The decrease on the relative abundance of *Streptococcus* may be related to the ability of rumen protozoa to prevent acidosis by engulfment of starch granules, promoting a more stable pH [60].

In this experiment we have shown that eighteen of the most abundant OTUs showed significant differences during successive colonization of the rumen by protozoa (Table S2). However almost 50% of them could not be classified beyond the level of class and three could not be identified at even phyla level. If the full advantages of NGS are to realised there is clearly a need for projects such as the Hungate 1000 (www.hungate1000.org.nz) which aim to characterise and sequence the genomes of the great many bacteria in the rumen that to date remain uncharacterised and indeed in many cases uncultured [61].

## Conclusions

Ion Torrent PGM is a reliable and cost-effective tool to study microbial diversity in complex ecosystems which compared well in terms of derived information to T-RFLP, especially when a high number of samples are to be studied. Furthermore, the additional information provided by the NGS data in terms of microbial classification that could be very important in studies focused in discovering key species affected by dietary or environmental shifts [45], [62]. However, our findings demonstrated that traditional fingerprinting methods, such as T-RFLP, give similar results to NGS and they can therefore provide still valuable information when NGS is not available or a non-targeted microbial analysis is required. Successive colonisation of the rumen by protozoa influenced the bacterial population present with increases in the relative abundances of *Prevotella*, *Bacteroides* and *Ruminobacter*. A more homogeneous bacterial community is observed when protozoa are present in the rumen, suggesting that rumen protozoa might help stabilise the rumen fermentation by reducing the variability in bacteria present among individual animals.

## Supporting Information

Figure S1 **Rarefaction curves. Genomic DNA was obtained from rumen samples from 8 animals either protozoa-free (P1), faunated with holotrich protozoa (P2) o with a complete protozoal population (P3).**
(TIF)

Table S1 **Barcode primers used for multiplexed Ion Torrent sequencing.**
(DOCX)

Table S2 **Classification of OTUs (18 of 864) found to shift in their relative abundance when progressive colonization of rumen protozoa is applied.** Samples come from rumen fluid from protozoa-free (P1), faunated with holotrich protozoa (P2) o with a complete protozoal population (P3) animals. Only OTUs with an average abundance of 0.01% or higher were considered.
(DOCX)
