# Supplementary figures and images for: Pros and Cons of Ion-Torrent Next Generation Sequencing versus Terminal Restriction Fragment Length Polymorphism T-RFLP for Studying the Rumen Bacterial Community
Source: PLoS One. 2014 Jul 22;9(7):e101435. doi: 10.1371/journal.pone.0101435 (PMC4106765; doi:10.1371/journal.pone.0101435)

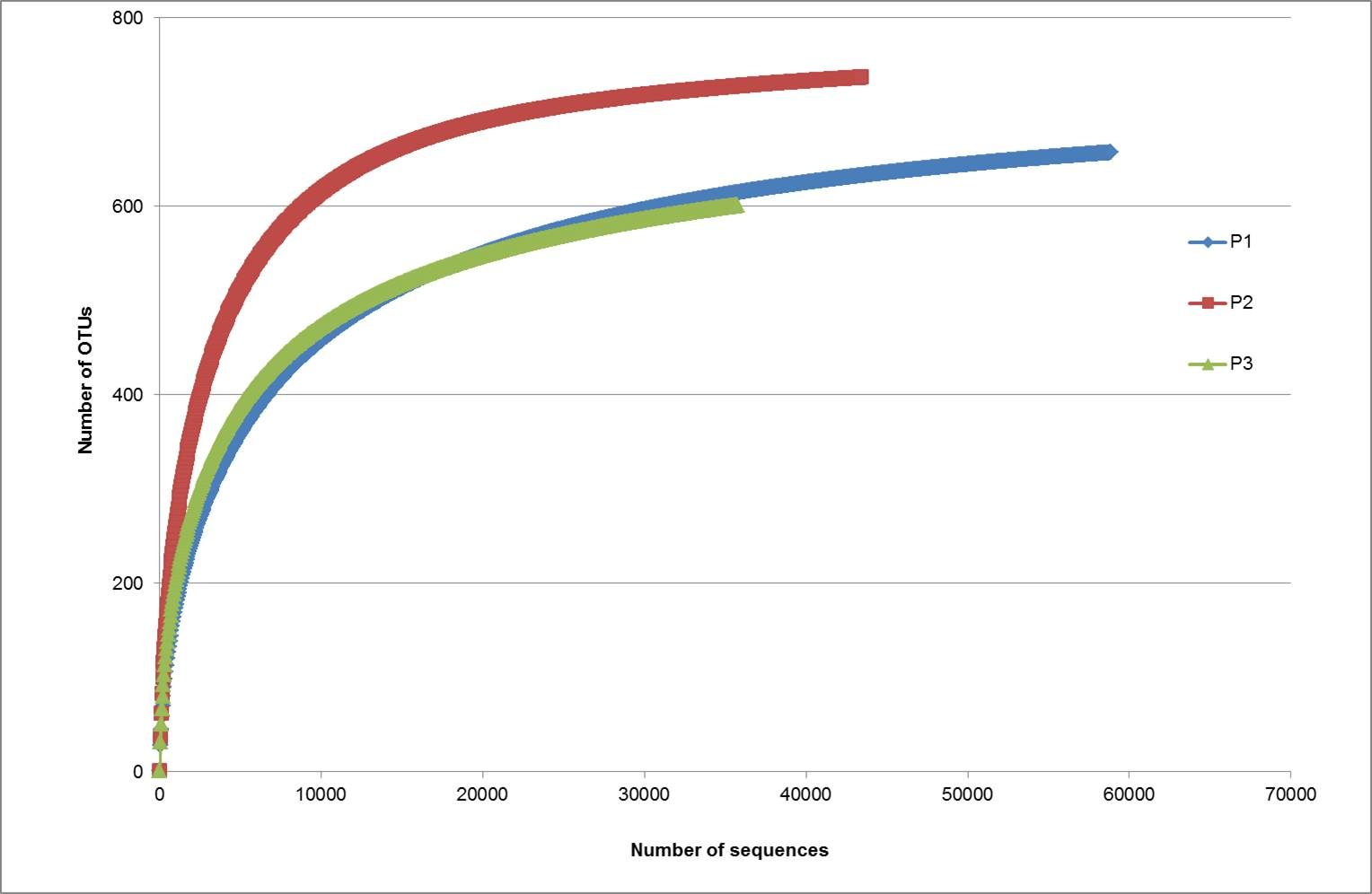

Supplement: Figure S1 — Rarefaction curves. Genomic DNA was obtained from rumen samples from 8 animals either protozoa-free (P1), faunated with holotrich protozoa (P2) o with a complete protozoal population (P3). (TIF) [file pone.0101435.s001.tif]
