# Supplementary material for: Pros and Cons of Ion-Torrent Next Generation Sequencing versus Terminal Restriction Fragment Length Polymorphism T-RFLP for Studying the Rumen Bacterial Community
Source: PLoS One. 2014 Jul 22;9(7):e101435. doi: 10.1371/journal.pone.0101435 (PMC4106765; doi:10.1371/journal.pone.0101435)
Supplement: Table S1 — Barcode primers used for multiplexed Ion Torrent sequencing. (DOCX) [file pone.0101435.s002.docx]

**Table S1** Barcode primers used for multiplexed Ion Torrent sequencing

| **Primer Name** | **Forward primer (Primer A-key) (30)** | **Ion Barcode (12)** |  | **Template specific sequence-3' (17)** |
| --- | --- | --- | --- | --- |
| PGM_V3_1 | CCATCTCATCCCTGCGTGTCTCCGACTCAG | GATCTGCGATCC | GT | CCTACGGGAGGCAGCAG |
| PGM_V3_2 | CCATCTCATCCCTGCGTGTCTCCGACTCAG | CAGCTCATCAGC | GT | CCTACGGGAGGCAGCAG |
| PGM_V3_3 | CCATCTCATCCCTGCGTGTCTCCGACTCAG | CAAACAACAGCT | GT | CCTACGGGAGGCAGCAG |
| PGM_V3_4 | CCATCTCATCCCTGCGTGTCTCCGACTCAG | GCAACACCATCC | GT | CCTACGGGAGGCAGCAG |
| PGM_V3_5 | CCATCTCATCCCTGCGTGTCTCCGACTCAG | GCGATATATCGC | GT | CCTACGGGAGGCAGCAG |
| PGM_V3_6 | CCATCTCATCCCTGCGTGTCTCCGACTCAG | CGAGCAATCCTA | GT | CCTACGGGAGGCAGCAG |
| PGM_V3_7 | CCATCTCATCCCTGCGTGTCTCCGACTCAG | AGTCGTGCACAT | GT | CCTACGGGAGGCAGCAG |
| PGM_V3_8 | CCATCTCATCCCTGCGTGTCTCCGACTCAG | GTATCTGCGCGT | GT | CCTACGGGAGGCAGCAG |
| PGM_V3_9 | CCATCTCATCCCTGCGTGTCTCCGACTCAG | CGAGGGCCCGTC | GT | CCTACGGGAGGCAGCAG |
| PGM_V3_10 | CCATCTCATCCCTGCGTGTCTCCGACTCAG | CAAATTCGGCAT | GT | CCTACGGGAGGCAGCAG |
| PGM_V3_11 | CCATCTCATCCCTGCGTGTCTCCGACTCAG | AGATTGACCAAC | GT | CCTACGGGAGGCAGCAG |
| PGM_V3_12 | CCATCTCATCCCTGCGTGTCTCCGACTCAG | AGTTACGAGCTA | GT | CCTACGGGAGGCAGCAG |
| PGM_V3_13 | CCATCTCATCCCTGCGTGTCTCCGACTCAG | GCATATGCACTG | GT | CCTACGGGAGGCAGCAG |
| PGM_V3_14 | CCATCTCATCCCTGCGTGTCTCCGACTCAG | CAACTCCCGTGA | GT | CCTACGGGAGGCAGCAG |
| PGM_V3_15 | CCATCTCATCCCTGCGTGTCTCCGACTCAG | TTGCGTTAGCAG | GT | CCTACGGGAGGCAGCAG |
| PGM_V3_16 | CCATCTCATCCCTGCGTGTCTCCGACTCAG | TACGAGCCCTAA | GT | CCTACGGGAGGCAGCAG |
| PGM_V3_17 | CCATCTCATCCCTGCGTGTCTCCGACTCAG | CACTACGCTAGA | GT | CCTACGGGAGGCAGCAG |
| PGM_V3_18 | CCATCTCATCCCTGCGTGTCTCCGACTCAG | TGCAGTCCTCGA | GT | CCTACGGGAGGCAGCAG |
| PGM_V3_19 | CCATCTCATCCCTGCGTGTCTCCGACTCAG | ACCATAGCTCCG | GT | CCTACGGGAGGCAGCAG |
| PGM_V3_20 | CCATCTCATCCCTGCGTGTCTCCGACTCAG | TCGACATGTCTT | GT | CCTACGGGAGGCAGCAG |
| PGM_V3_21 | CCATCTCATCCCTGCGTGTCTCCGACTCAG | GAACACTTTGGA | GT | CCTACGGGAGGCAGCAG |
| PGM_V3_22 | CCATCTCATCCCTGCGTGTCTCCGACTCAG | CAGCCATCTGTA | GT | CCTACGGGAGGCAGCAG |
| PGM_V3_23 | CCATCTCATCCCTGCGTGTCTCCGACTCAG | TTGGGTACACGT | GT | CCTACGGGAGGCAGCAG |
| PGM_V3_24 | CCATCTCATCCCTGCGTGTCTCCGACTCAG | AAGGCGCTCCTT | GT | CCTACGGGAGGCAGCAG |
|  | **Reverse primer (Primer P1-key) (23)** |  |  |  |
| PGM_V3_Rev | CCTCTCTATGGGCAGTCGGTGAT | none | CC | ATTACCGCGGCTGCTGG |
