# Supplementary material for: Pros and Cons of Ion-Torrent Next Generation Sequencing versus Terminal Restriction Fragment Length Polymorphism T-RFLP for Studying the Rumen Bacterial Community
Source: PLoS One. 2014 Jul 22;9(7):e101435. doi: 10.1371/journal.pone.0101435 (PMC4106765; doi:10.1371/journal.pone.0101435)
Supplement: Table S2 — Classification of OTUs (18 of 864) found to shift in their relative abundance when progressive colonization of rumen protozoa is applied. Samples come from rumen fluid from protozoa-free (P1), faunated with holotrich protozoa (P2) o with a complete protozoal population (P3) animals. Only OTUs with an average abundance of 0.01% or higher were considered. (DOCX) [file pone.0101435.s003.docx]

**Table S2** Classification of OTUs (18 of 864) found to shift in their relative abundance when progressive colonization of rumen protozoa is applied. Samples come from rumen fluid from protozoa-free (P1), faunated with holotrich protozoa (P2) o with a complete protozoal population (P3) animals. Only OTUs with an average abundance of 0.01% or higher were considered

| **O.T.U.** | **Phyla** | **Class** | **Order** | **Family** | **Genus** | **ID** | **P1** | **P2** | **P3** | **P value** |
| --- | --- | --- | --- | --- | --- | --- | --- | --- | --- | --- |
| 2 | Bacteroidetes | Bacteroidia | Bacteroidales | Porphyromonadaceae | Unclassified | A1.P1_U5C2J:1023:1175 | 0.0399 | 0.0270 | 0.1497 | 0.029 |
| 10 | Bacteroidetes | Bacteroidia | Bacteroidales | Prevotellaceae | Unclassified | A1.P1_U5C2J:864:1164 | 0.0158 | 0.0271 | 0.0061 | 0.064 |
| 40 | SR1 | SR1_genera_incertae_sedis | Unclassified | Unclassified | Unclassified | A8.P1_JEQUT:1880:1104 | 0.0012 | 0.0028 | 0.0040 | 0.064 |
| 52 | Bacteroidetes | Bacteroidia | Bacteroidales | Prevotellaceae | Prevotella | A1.P3_U5C2J:1019:501 | 0.0002 | 0.0002 | 0.0271 | 0.029 |
| 61 | Bacteroidetes | Bacteroidia | Bacteroidales | Prevotellaceae | Prevotella | A4.P1_U5C2J:702:205 | 0.0073 | 0.0067 | 0.0003 | 0.076 |
| 64 | Bacteroidetes | Bacteroidia | Bacteroidales | Porphyromonadaceae | Unclassified | A1.P3_U5C2J:1036:1010 | 0.0000 | 0.0000 | 0.0073 | 0.036 |
| 67 | Unclassified | Unclassified | Unclassified | Unclassified | Unclassified | A3.P3_JEQUT:411:1915 | 0.0001 | 0.0001 | 0.0038 | 0.018 |
| 76 | Bacteroidetes | Bacteroidia | Bacteroidales | Prevotellaceae | Prevotella | A8.P2_U5C2J:570:181 | 0.0000 | 0.0004 | 0.0094 | 0.023 |
| 78 | Firmicutes | Unclassified | Unclassified | Unclassified | Unclassified | A3.P3_U5C2J:1137:911 | 0.0000 | 0.0001 | 0.0037 | 0.023 |
| 96 | Bacteroidetes | Bacteroidia | Bacteroidales | Porphyromonadaceae | Unclassified | A4.P1_U5C2J:1132:685 | 0.0011 | 0.0024 | 0.0009 | 0.064 |
| 105 | Bacteroidetes | Bacteroidia | Bacteroidales | Prevotellaceae | Unclassified | A2.P3_U5C2J:688:292 | 0.0017 | 0.0020 | 0.0001 | 0.065 |
| 144 | Unclassified | Unclassified | Unclassified | Unclassified | Unclassified | A6.P3_U5C2J:586:982 | 0.0056 | 0.0028 | 0.0013 | 0.076 |
| 154 | Bacteroidetes | Bacteroidia | Bacteroidales | Unclassified | Unclassified | A1.P1_U5C2J:35:188 | 0.0030 | 0.0007 | 0.0005 | 0.095 |
| 159 | Bacteroidetes | Bacteroidia | Bacteroidales | Porphyromonadaceae | Unclassified | A2.P1_U5C2J:39:555 | 0.0007 | 0.0006 | 0.0024 | 0.083 |
| 190 | Bacteroidetes | Unclassified | Unclassified | Unclassified | Unclassified | A2.P2_U5C2J:702:968 | 0.0001 | 0.0002 | 0.0027 | 0.023 |
| 203 | Bacteroidetes | Flavobacteria | Flavobacteriales | Flavobacteriaceae | Unclassified | A4.P2_U5C2J:387:24 | 0.0015 | 0.0020 | 0.0015 | 0.077 |
| 213 | Unclassified | Unclassified | Unclassified | Unclassified | Unclassified | A7.P3_JEQUT:1261:436 | 0.0000 | 0.0000 | 0.0062 | 0.018 |
| 255 | Firmicutes | Unclassified | Unclassified | Unclassified | Unclassified | A3.P2_U5C2J:381:552 | 0.0010 | 0.0017 | 0.0008 | 0.093 |
